# A Study on the Effects of a Self-Administered Eye Exercise Program on the Balance and Gait Ability of Chronic Stroke Patients: A Randomized Controlled Trial

**DOI:** 10.3390/jpm14060595

**Published:** 2024-06-02

**Authors:** Chung-Hyun Son, Geon-Woo Sim, Kyoung Kim

**Affiliations:** Department of Physical Therapy, College of Rehabilitation Science, Daegu University, Gyeongsan 38453, Republic of Korea; pok0055@naver.com (C.-H.S.); geonwoo510@nate.com (G.-W.S.)

**Keywords:** self-administered exercise, balance, gait, stroke, eye exercise

## Abstract

This study investigates the effects of a self-administered eye exercise (SEE) program on the balance and gait ability of chronic stroke patients hospitalized due to hemiplegia. This study includes 42 patients diagnosed with stroke-related hemiplegia and hospitalized at D Rehabilitation Hospital. The researcher randomly allocated 42 patients into two groups: the experimental group (EG, *n* = 21, mean age = 58.14 ± 7.69 years, mean BMI = 22.83 ± 2.19 kg/m^2^) and the control group (CG, *n* = 21, mean age = 58.57 ± 6.53 years, mean BMI = 22.81 ± 2.36 kg/m^2^). The SEE program was applied to the EG and the general self-administered exercise (SE) program was applied to the CG. After 4 weeks of intervention, weight distribution of the affected side, the Timed Up and Go test (TUG), step length of the affected side, step length of the unaffected side, gait speed, and cadence were analyzed and compared. In the within-group comparison, both groups showed significant differences in weight distribution (*p* < 0.05), TUG (*p* < 0.05), step length of the affected side (*p* < 0.05), step length of the unaffected side (*p* < 0.05), gait speed (*p* < 0.05), and cadence (*p* < 0.05). In the between-group comparison, a significant difference in the TUG (*p* < 0.05) was observed. The SEE program had an overall similar effect to the SE program in improving the balance and gait ability of chronic stroke patients, and had a greater effect on dynamic balance ability. Therefore, the SEE program can be proposed as a self-administered exercise program to improve balance and gait ability in stroke patients who are too weak to perform the SE program in a clinical environment or have a high risk of falling.

## 1. Introduction

Stroke is a serious neurological disease that occurs in about 17 million people every year [1]. It is the second-highest cause of death in the world [2]. Stroke is a lesion in which brain function is partially damaged due to a blockage or bleeding of the cerebrovascular vessels, resulting in various symptoms [3]. After a stroke, 75% of patients complain of neurological dysfunction, including unilateral paralysis of the opposite side of the injury, decreased visual perception and cognitive function, and decreased balance and gait ability [4,5]. Impaired dysfunction improves for the first 3 months after a stroke and then worsens, which is why long-term therapeutic exercise is necessary [4]. The increasing prevalence of stroke and the necessity for long-term care is a financial burden on individual patients [6]. The importance of rehabilitation, such as the prevention of secondary disabilities and cost-effective strategies for stroke patients, is being emphasized [7].

Balance is divided into static balance and dynamic balance; static balance refers to the ability to stand on a base of support without body agitation, and dynamic balance refers to the ability to move against a moving base of support or other external stimulus [8]. Balance ability is a mechanism controlled by the integration of somatosensory, vestibular, and visual information [9]. However, when an imbalance occurs due to stroke, body control and gaze movement are reduced, which leads to a decrease in gait ability [10,11]. For example, when walking straight, the head movement and gaze motion increase abnormally, which is a result of defects in the stabilization mechanisms [12]. This imbalance leads to problems such as a longer swing phase time and shorter single-limb support time of the affected lower extremities, resulting in a decrease in gait speed [12,13].

The eye exercise program consists of vestibulo-ocular reflex (VOR), smooth pursuit, optokinetic, vergence, fixation, and saccade exercises [14]. The vestibular system contributes to visual stabilization through the VOR [15]. The VOR stabilizes the eye position in space (gaze stabilization) during head movements, producing eye movement at equal speed and in the opposite direction to the head movement to allow for proper vision [16]. An example of horizontal plane movement is when the vestibular organ reacts to the left head rotation and fixes the eye through the contraction of the medial rectus of the left eye and the lateral rectus of the other eye [17]. The VOR exercise is used because sensory conflict leads to neurological rearrangements through vestibular compensation [18]. Smooth pursuit refers to smooth eye movement, which is commonly used to maintain the gaze on an object regardless of whether it is stationary or moving [19]. Optokinetic exercise refers to the movement of the eye necessary to view the entire visual scene during continuous self-rotation, and vergence exercise refers to the movement that allows the individual to ascertain whether an object is moving closer or farther away [20]. Fixation is a movement that occurs to fix the eye by identifying and correcting the image formed on the retina from visual information so that involuntary and unnecessary saccades do not occur [21]. The saccade exercise is used to move the eye toward a visual target, move the eye to the location that the target used to occupy, or anticipate or identify the appearance of a target in a specific location [22].

Eye exercises are divided into gaze stability exercises and gaze shift exercises in terms of function [14]. Among them, gaze stability exercises are further divided into adaptation and substitution exercises [23]. Adaptation exercises facilitate long-term changes in neurological responses to reduce abnormal symptoms and improve gaze and balance ability, and substitution exercises are used to develop strategies such as smooth pursuit or central pre-programming [24]. Gaze stability exercises are also known as habituation exercises, which are thought to habituate related symptoms by repeated exposure to stimuli to reduce the response to abnormal stimuli [25]. Previous studies have suggested that the eye exercise program is easy and simple, so, after supervised training, it can be performed as a self-administered exercise [26,27]. In other studies, the eye exercise program was effective in restoring the balance and gait ability of stroke patients [28], and eye exercise including gaze stabilization has a certain effect on the recovery of impaired balance and gait ability after stroke [12]. In addition, vestibular rehabilitation therapy, including the eye exercise program, showed positive effects on the gait ability of stroke patients [29].

The higher the intensity of therapeutic activity in recovery after damage to the nervous system, such as stroke, the more effective it is [30]. In addition, it is reported that the earlier and more focused the rehabilitation, the better the functional recovery [31]. It is recommended that the duration of therapeutic activities during the day should be long for effective treatment intervention, and that additional exercise programs other than treatment times are required. However, there is a limitation in that most treatments are only provided for a certain amount of time in the hospital, so various self-administered exercise programs are being proposed to improve the balance and gait ability of stroke patients [32]. Galvin et al. reported that additional exercise programs on the lower extremities had a significant effect on gait speed and emphasized the importance of additional exercise programs for functional improvement in stroke patients [33]. The Otago exercise program, which consists of balance exercises, walking exercises, and muscle strengthening exercises, is reported as an effective self-administered exercise program to restore the balance and gait ability in older people [34]. Previous studies have confirmed that eye exercise can improve balance and gait ability in stroke patients [12,28,29] and have also confirmed that inpatients need several self-administered exercise programs for their recovery [32,33,34]. However, there is a lack of research on whether eye exercise has a positive effect on balance and gait ability by implementing the eye exercise program in chronic stroke patients as a self-administered approach. The purpose of this study is to compare a group of chronic stroke patients admitted to hospital who were given the self-administered eye exercise program with a group who were given the general self-administered exercise program to determine how the self-administered exercise program affects the balance and gait ability of inpatients.

## 2. Methods

### 2.1. Participants

The study was conducted according to the guidelines of the Declaration of Helsinki and approved by the Institutional Review Board of Daegu University (IRB no. 1040621-202301-HR-015). The randomized controlled trial was registered in the Clinical Research Information Service, and the Registration Number is KCT0009490. Patients with stroke who were hospitalized at D Rehabilitation Hospital in Daegu, Republic of Korea, participated in this study. G-power 3.1.9.7 was used to determine the number of participants. To calculate the number of participants, the effect size of the cadence value among the gait ability variables was taken from a similar type of previous study that included eye exercises. When the data after 4 weeks of the experiment were compared between groups, the effect size was 0.89 [29]. A total of 42 participants were calculated as an effect size d of 0.89, a significance level of 0.05, and a power of 80% (1-β err prob). An additional 10% was added to account for the dropout rate, bringing the total number of participants to 46.

The criteria for selecting participants were as follows: patients with hemiplegia due to a diagnosis of stroke, but could walk more than 3 m (with or without assistance) [12]; patients who had a stroke more than 6 months and less than 24 months ago; patients who could follow and perform instructions well with an MMSE-K of 24 points or higher; and patients who had undergone injections of Botulinum toxin type A. The exclusion criterion was patients with neurological conditions other than stroke [35].

### 2.2. Experimental Procedure

This study was conducted according to the Consolidated Standards of Reporting Trials (CONSORT) guidelines [36] and used a prospective, single-blinded, controlled, randomized design in which the participants were randomly assigned to either the experimental group (EG) or the control group (CG). Participants were recruited for 2 weeks and selected according to the inclusion and exclusion criteria for 1 week. After that, 1 week was assigned for the pre-experimental test and 1 week was assigned for the post-experimental test. Before the start of the experiment, informed consent was obtained from all participants involved in the study. Homogeneity among participants was secured through face-to-face interviews with nursing information surveys collecting data such as age, gender, weight, height, diagnosis, and duration. For randomization, a draw with A or B was prepared under the supervision of a chief investigator in the hospital, and A was assigned as an EG (n = 23) and B as a CG (n = 23). However, a total of 21 participants were assigned to the EG (mean age = 58.14 ± 7.69 years, mean BMI = 22.83 ± 2.19 kg/m^2^) and 21 to the CG (mean age = 58.57 ± 6.53 years, mean BMI = 22.81 ± 2.36 kg/m^2^), with 2 participants refusing to participate and 2 leaving the hospital.

**Figure 1:**
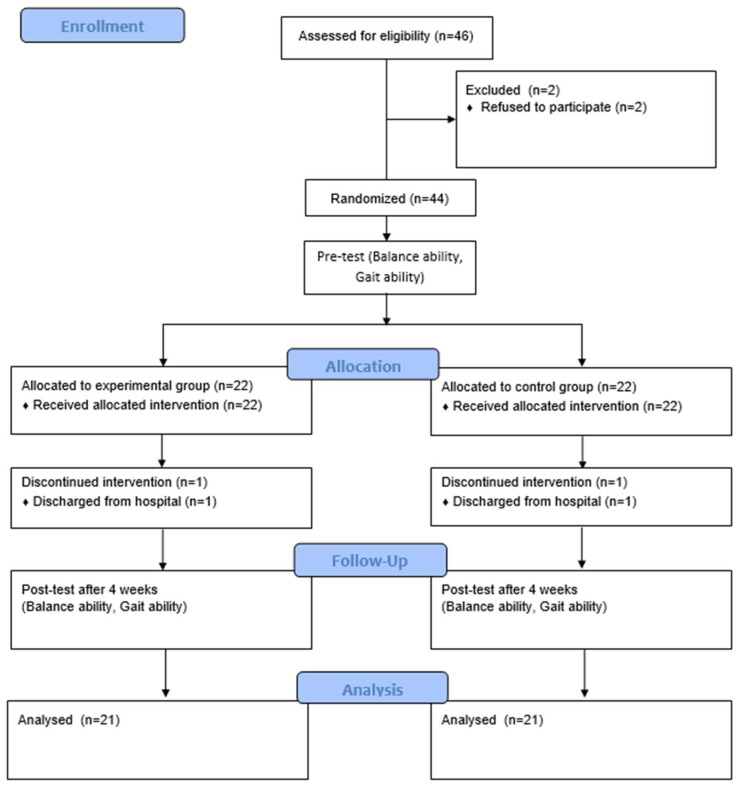
Study flow chart.

Both the self-administered eye exercise (SEE) program and general self-administered exercise (SE) program were assigned to the EG, and the SE program was assigned to the CG. Both groups exercised for 30 min a day, 5 times a week, for 4 weeks [24]. After the participants had been trained in each program by a therapist, exercise guidelines were provided. To check whether the exercises had been implemented, a table was prepared to record whether they had been completed and the date, and the resident rehabilitation support staff were asked to encourage participation in the self-administered exercises (Figure 1).

### 2.3. Self-Administered Eye Exercise Program

The SEE program was reported using the Template for Intervention Description and Replication (TIDieR) guidelines [37] and was modified in previous studies [38,39]. The composition of the program is shown in Table 1.

### 2.4. General Self-Administered Exercise Program

The general SE program was modified in previous studies. SE program is conducted for 15 min in EG and 30 min in CG, including quick rest. Each exercise was performed 10 to 15 times repeatedly. The composition of the program is shown in Table 2.

### 2.5. Balance Ability Measurement

#### 2.5.1. Gait Checker

Gait Checker (GhiWell Co., Ltd., Yangju, Republic of Korea) is a test for measuring static balance ability, and was used in this study. It collects data using a pressure sensor, transmits it to a computer through a USB cable, and analyzes it by a measuring device. The collected data include the weight distribution of the affected side (%). All participants were measured for 30 s with their eyes staring straight ahead at the Gait Checker, and the therapist stood next to the participant to prevent falls. This test was carried out 3 times, and the average value was used.

#### 2.5.2. Timed Up and Go Test

The Timed Up and Go test (TUG) is a test for measuring dynamic balance ability. The TUG test starts with the participant in a sitting position in the chair; they get up after hearing the start signal, walk 3 m, turn around the turning point, walk back, and sit down in the chair. The test is conducted three times and the results are averaged [40]. The TUG has high test–retest reliability and inter-scorer reliability, with ICCs of 0.95 and 0.98 each [41].

### 2.6. Gait Ability Measurement

#### GAITRite System

GAITRite (CIR Systems, Clifton, NJ, USA) is a thin rubber mat with built-in pressure-sensing devices placed at 10 mm intervals, and is a system that automatically records the time and position of the foot falling. The collected data are gait speed (cm/s), the number of steps per minute (step/min), and the length of step (cm). The participants were positioned 2 m away from the analyzer and instructed to walk at a comfortable speed. This test was conducted 3 times, and the average value was used. GAITRite has high test–retest reliability, with an ICC of 0.72~0.94 [42].

### 2.7. Data Processing

#### 2.7.1. Statistical Analysis

Data analysis was carried out using SPSS for Windows version 23.0 software (IBM Corp., Armonk, NY, USA). All data were described as mean ± standard deviation. Shapiro–Wilk’s test was performed to confirm the normality distribution, and all data showed a normal distribution. An independent *t*-test was performed to test the homogeneity of the experimental group and the control group. A paired *t*-test was performed to compare the pre-intervention and post-intervention data within the two groups. An independent *t*-test was also performed to compare the pre-intervention and post-intervention data between the two groups. The statistical significance level (α) was set to 0.05.

#### 2.7.2. Cohen’s d

Cohen’s d formula was used for the effect size equivalent to the effects noticed for within-group and between-group comparisons of the two groups. We analyzed it with G-power 3.1.9.7 using the mean, SD, and correlation coefficient. An effect size d of 0.2 was interpreted as “low”, 0.5 as “average”, and 0.8 as “strong” [43].

#### 2.7.3. Statistical Power

Post hoc power analysis was used to confirm the power between groups with significant differences. The effect size d, the statistical significance level of 0.05, and the sample size of the two groups were analyzed using G-power 3.1.9.7 [44].

## 3. Results

### 3.1. Baseline Characteristics of the Participants

No significant difference in the baseline characteristics was observed between the two groups (*p* > 0.05; Table 3).

### 3.2. Comparison of Balance Ability within and between Groups

There were significant differences in the balance ability after the intervention in both groups (*p* < 0.05).

In the comparison between groups, there was no difference in the starting point between the groups before the intervention. After the intervention, there was a significant difference in the TUG between the groups (*p* < 0.05, effect size *d* = 0.650, statistical power = 0.538), but there was no significant difference in the comparison of weight distribution of the affected side (*p* > 0.05) (Table 4).

### 3.3. Comparison of Gait Ability within and between Groups

There were significant differences in the gait ability after intervention in both groups (*p* < 0.05).

In the comparison between groups, there was no difference in the starting point between groups before the intervention. After the intervention, there was no significant difference in gait ability between the groups (*p* > 0.05) (Table 5).

## 4. Discussion

This study was conducted to investigate the effect of a self-administered eye exercise program on the balance and gait ability of chronic stroke patients. Both the SEE and SE groups received a traditional stroke rehabilitation program and were additionally assigned to a self-administered exercise program. The SEE group performed both a self-administered eye exercise program and a general self-administered exercise program, and the SE group performed only a general self-administered exercise program. The results of the study showed that balance and gait ability improved in both the SEE and SE groups compared to before the intervention. Although the SEE group showed greater improvement compared to the SE group in dynamic balance ability, there were no significant differences between the two groups in terms of static balance ability and gait ability. These results provide evidence that a self-directed eye exercise program has a positive effect on the dynamic balance ability of stroke patients.

In this study, the balance ability of chronic stroke patients improved in both the SEE and SE groups. In addition, the SEE group’s self-administered eye exercise program improved dynamic balance ability to a greater extent than the SE group’s general self-administered exercise program. In this study, dynamic balance ability was evaluated using the TUG. Previous studies have shown that gaze stabilization exercises, including VOR, and saccadic exercises can improve dynamic balance by significantly reducing the TUG in acute stroke patients [12]. Another study reported that a 3-week home program consisting of eye movement and gaze stabilization exercises reduced the TUG in stroke patients and significantly lowered the fall risk estimate [45]. In this study, the TUG was significantly decreased in the SEE group compared to the SE group, showing the same results as previous studies. It is said that visual stimulation is transmitted through the central nervous system and integrated with other sensory stimulation to enable the correct predictive control of muscles [46]. In addition, it is said that adaptation of the vestibular system through VOR exercise contributes to the improvement in dynamic balance ability [47]. VOR is most important in gaze stability [48]. Gaze stability is necessary to control the movements of the head, trunk, and pelvis during movement [49]. In this study, it is believed that the self-administered eye exercise program induced normal eye movement and activated the VOR, thereby promoting the interaction between vestibular function and vision and improving postural control and stability, resulting in a greater improvement in dynamic balance ability.

Previous studies have reported that an eye exercise program consisting of saccadic movements, smooth pursuit movements, and adaptive movements is effective in improving the static balance ability of stroke patients [50]. It was also found that an eye exercise program including saccades, smooth pursuit, VOR, and convergence exercises improved the balance ability of elderly people [39]. Additionally, it was reported that smooth pursuit and saccadic exercises stimulate eye muscles and improve static balance ability [51]. In this study, the self-administered eye exercise program applied to the SEE group improved static balance ability, showing the same results as previous studies. However, static balance ability also improved in the SE group, and there was no significant difference between the two groups. This is thought to be because the exercise method included in the general self-administered exercise program strengthens the trunk muscles and improves static balance ability. Stroke patients have reduced balance due to damage to their trunk muscles [52]. Previous studies have also reported that additional trunk stabilization exercises have a positive effect on improving balance in subacute stroke patients [53]. Balance is the biggest factor affecting the function of stroke patients [54]. The results of this study showed that the self-administered eye exercise program improved both static and dynamic balance abilities, and was more effective in improving dynamic balance ability than the general self-administered exercise program. Therefore, to improve the balance ability of stroke patients, a self-administered eye exercise program can be proposed after traditional stroke rehabilitation treatment.

In this study, the gait ability of the chronic stroke patients in the SEE group improved. Stroke patients’ gait is an important factor in determining daily life activities and prognosis [55] and is an important measure of motor function and recovery [56]. Many studies have reported that eye exercise programs improve the gait ability of stroke patients. One study showed that vestibular rehabilitation treatment, including eye exercises, could improve spatiotemporal gait parameters and make gait safer in stroke patients [29]. This study reported that vestibular rehabilitation treatment that included eye exercises improved postural control, weight distribution, and direction during gait in stroke patients. Another study reported that vestibular rehabilitation treatment including gaze stabilization exercises had a positive effect on the gait ability of acute stroke patients [57]. There is also a study that reported that an eye exercise program using cards and tools improved retaliation, gait speed, and cadence in stroke patients [58]. This study showed that gait can be improved if the visual and vestibular organs are directly stimulated. A study investigating the effects of eye movement and visual perception training on stroke patients found that it significantly improved gait speed [59]. Additionally, there is a study that reported that vestibular rehabilitation treatment including gaze stabilization exercises improved the VOR of stroke patients, leading to improved gait performance [60]. As such, many previous studies have reported that eye exercise improves various gait parameters, supporting the results of this study. It is thought that the self-administered eye exercise program applied in this study improved spatial orientation and postural stability when walking by causing various eye movements when the positions of the upper limbs and trunk changed. In addition, the improved dynamic balance ability found in this study is thought to have had a positive effect on gait.

Gait ability also improved in the SE group and was not significantly different from the SEE group. It is thought that the exercise method included in the general self-administered exercise program strengthened the trunk muscles and had a positive effect on gait ability. In a previous study, it was reported that gait ability was significantly improved by applying trunk stabilization exercises to stroke patients for 6 weeks [61]. Additionally, a systematic review study showed that trunk training improves trunk control and mobility in stroke patients [62]. In this study, it is believed that the SE program applied along with the traditional stroke rehabilitation program improved the participants’ trunk performance and balance ability, thereby improving their gait ability.

This study has several limitations. First, because the study targeted patients with chronic stroke, additional research targeting patients with acute stroke within 6 months of onset is needed. Second, the SEE program did not define the distance between the eye and the second finger. Third, because there was no follow-up observation, it was not possible to confirm how effective the SEE program was maintained. Fourth, because both the SEE and SE groups were given self-administered exercise programs, it was not possible to control for all possible confounding variables.

## 5. Conclusions

The SEE program had an overall similar effect to the SE program in terms of improving the balance and gait ability of chronic stroke patients, and had a greater effect on dynamic balance ability. Therefore, the SEE program can be proposed as a self-administered exercise program to improve balance and gait ability in stroke patients who are too weak to perform the SE program in a clinical environment or have a high risk of falling.

## Figures and Tables

**Table 1:** Description of the SEE program.

Intervention Description and Replication (TIDieR) Guidelines	
Name	The SEE program.
Why	To enhance balance and gait ability of chronic stroke patients (EG, n = 21).
Materials	The SEE program guideline paper, trained by a therapist.
Procedures	The SEE program consisted of 5 types of programs and was implemented 15 min a day (including quick break), 5 times a week, and 4 weeks. VOR exercise: In a sitting position, extend your arms, stretch your second finger, and stare at your finger with your head fixed. Keep your finger fixed, staring with the eyes, and move your head left and right, up and down. VOR adaptation exercise: In a sitting position, extend your arms, stretch your second finger, and stare at your finger with your head fixed. Eyes stare at the finger and move your fingers and head in the opposite left and right directions, respectively, or in the opposite direction of up and down. Smooth pursuit and optokinetic exercise: In a sitting position, extend your arms, stretch your second finger, and stare at your finger with your head fixed. Move your finger and head smoothly in the same direction as left and right, respectively, or smoothly in the same direction as up and down. Vergence exercise: In a sitting position, extend your arms, stretch your second finger, and stare at your finger with your head fixed. Keep your finger close to your eyes and away from your eyes. Saccade exercise: In a sitting position, stare at the point in the middle. With the head fixed, look at the left target, stare at the center point again, look at the right target, stare at the center point again, look at the upper target, stare at the center point again, look at the lower target, stare at the center point again.
Who	Physical therapist with extensive treatment experience who took SEE program-related training and Vestibular Rehabilitation Therapy (VRT) specialized training.
How	Self-administered exercise, individually.
Where	In a hospital room.
How much	15 min a day (including quick break), 5 times a week, and 4 weeks.
Tailoring	The frequency, time, and duration of the SEE program were determined by referring to the study of Hall et al. [24].
Modifications	Modified and supplemented the study of Pimenta et al. [38], Roh and Lee [39].
How planned Actual	Prepare an experimental participation record sheet for successful intervention complete and asked the resident rehabilitation support staff to encourage participation. All participants completed the SEE program.

**Table 2:** General self-administered exercise program.

Exercise	
Hip-lifting exercise	In a hook-lying position, both feet are shoulder-width apart. Perform the hip lift.
Sit to stand exercise	In a sitting position, both feet are shoulder-width apart and hold the fixed support to the unaffected side. Perform standing up.
One step exercise	In a standing position, both feet are shoulder-width apart and hold the fixed support to the unaffected side. Put one step forward and stand back again. Do it alternately with both feet.

**Table 3:** Baseline characteristics of the participants.

Variable	EG (n = 21)	CG (n = 21)	t (*p*)
Age (year)	58.14 ± 7.69	58.57 ± 6.53	−0.195 (0.847)
Sex			
Male	13	13	
Female	8	8	
Height (cm)	167.33 ± 8.45	166.38 ± 8.94	0.355 (0.725)
Weight (kg)	63.90 ± 7.53	63.52 ± 10.65	0.104 (0.894)
BMI (kg/m^2^)	22.83 ± 2.19	22.81 ± 2.36	0.020 (0.418)
Duration (month)	13.90 ± 4.10	13.43 ± 3.60	0.400 (0.578)
Weight distribution (affected side, %)	43.67 ± 2.80	43.81 ± 3.16	−0.155 (0.877)
TUG (s)	23.46 ± 5.08	23.40 ± 7.00	0.031 (0.976)
Step length (affected side, cm)	35.50 ± 8.24	35.07 ± 7.02	0.179 (0.859)
Step length (unaffected side, cm)	27.06 ± 8.34	28.19 ± 9.83	−0.401 (0.691)
Gait speed (cm/s)	41.43 ± 16.98	43.39 ± 14.64	−0.400 (0.691)
Cadence (step/min)	72.05 ± 18.80	68.70 ± 21.50	0.538 (0.594)

EG: experimental group, CG: control group.

**Table 4:** Comparison of balance ability within and between groups.

Variable	Group	Pre	Post	Difference Value	Within-Group		Between-Group	
					t	*p* (*d*)	t	*p* (*d*)
Weight distribution (affected side, %)	EG (n = 21)	43.67 ± 2.80	48.76 ± 1.45	5.10 ± 2.74	−8.531	<0.001 ^‡^ (2.283)	0.740	0.464
	CG (n = 21)	43.81 ± 3.16	48.24 ± 1.00	4.43 ± 3.09	−6.585	<0.001 ^‡^ (1.890)		
Timed Up and Go test (s)	EG (n = 21)	23.46 ± 5.08	18.83 ± 4.48	−4.62 ± 3.23	6.569	<0.001 ^‡^ (0.967)	−2.111	0.041 * (0.650)
	CG (n = 21)	23.40 ± 7.00	20.56 ± 5.70	−2.84 ± 2.14	6.096	<0.001 ^‡^ (0.445)		

EG: experimental group, CG: control group. * *p* < 0.05 significant in independent *t*-test, ^‡^ *p* < 0.05 significant in paired *t*-test, *d*: effect size d.

**Table 5:** Comparison of gait ability within and between groups.

Variable	Group	Pre	Post	Difference Value	Within-Group		Between-Group	
					t	*p* (*d*)	t	*p* (*d*)
Step length (affected side, cm)	EG (n = 21)	35.50 ± 8.24	40.86 ± 11.31	5.36 ± 8.60	−2.857	0.010 ^‡^ (0.542)	−0.342	0.734
	CG (n = 21)	35.07 ± 7.02	41.40 ± 10.79	6.33 ± 9.68	−2.997	0.007 ^‡^ (0.695)		
Step length (unaffected side, cm)	EG (n = 21)	27.06 ± 8.34	36.27 ± 11.07	9.21 ± 6.51	−6.483	<0.001 ^‡^ (0.940)	0.005	0.996
	CG (n = 21)	28.19 ± 9.83	37.39 ± 12.66	9.20 ± 6.70	−6.296	<0.001 ^‡^ (0.812)		
Gait speed (cm/s)	EG (n = 21)	41.43 ± 16.98	58.98 ± 22.49	17.55 ± 11.63	−6.912	<0.001 ^‡^ (0.881)	0.822	0.416
	CG (n = 21)	43.39 ± 14.64	58.27 ± 16.68	14.88 ± 9.25	−7.374	<0.001 ^‡^ (0.948)		
Cadence (step/min)	EG (n = 21)	72.05 ± 18.80	82.81 ± 19.38	10.76 ± 7.62	−6.475	<0.001 ^‡^ (0.564)	0.798	0.429
	CG (n = 21)	68.70 ± 21.50	77.41 ± 18.86	8.72 ± 8.91	−4.181	<0.001 ^‡^ (0.431)		

EG: experimental group, CG: control group. ^‡^ *p* < 0.05 significant in paired *t*-test, *d:* effect size d.

## Data Availability

The data presented in this study are available on request from the corresponding author. The data are not publicly available due to ethical restrictions.
